# Enhanced Vestibulo-Ocular Reflex Responses on vHIT. Is It a Casual Finding or a Sign of Vestibular Dysfunction?

**DOI:** 10.3389/fneur.2018.00866

**Published:** 2018-10-15

**Authors:** Jorge Rey-Martinez, Ann M. Burgess, Ian S. Curthoys

**Affiliations:** ^1^Otoneurology Unit, Otolaryngology Department, Hospital Universitario Donostia, San Sebastian, Spain; ^2^Vestibular Research Laboratory, School of Psychology, The University of Sydney, Sydney, NSW, Australia

**Keywords:** vHIT, hydrops, Meniere disease, sign, enhanced eye velocity

## Abstract

In current clinical practice, when in response to vHIT testing the observed slow-phase eye-velocity responses are significantly higher than head velocity, the most probable cause is considered to be an inadequate collection method or a recording artifact. We present two cases with clinical diagnoses of Menière's Disease: for both cases, enhanced eye velocity responses were measured with a rigorous vHIT testing protocol. In the first case we measured these enhanced responses on each test performed during a 5 year time series; in the second case multiple measurements were obtained from a patient after the radiologic diagnosis of vestibulo-cochlear hydrops. The two cases presented and the new evidence reported by other researchers suggest that owing to the low probability of artifact and the high consistency of the vHIT measurements, we should consider the hypothesis of a physio-pathologic cause for the enhanced eye responses to vHIT testing of some patients with vestibular dysfunction.

## Background

The video head impulse test (vHIT) is an objective computer-based clinical test of semicircular canal function that has a wide application for clinical pathologies where many vestibular and central disorders are involved (1). vHIT has two main objective outputs to be considered on clinical practice. The first is the mathematical relation between eye and head velocity during the slow phase period (1) known as the gain of the angular vestibulo-ocular reflex (aVOR) (2), and the second is the timing, grouping and velocity characteristics of the saccadic eye responses produced during slow- or fast-phase periods (3).

It has been also widely accepted that the presence of a lower value of aVOR gain—corresponding to situations where the slow-phase eye velocity is lower than head velocity most of the time—is a direct indicator of vestibular hypofunction (1).

At present, there is no evidence of other kinds of alteration of head and eye velocity relationships with clinical significance using vHIT. In this case report we present two clinical cases in which eye velocity was consistently found to be enhanced in relation to head velocity, to ask whether this enhancement is a direct indicator of peripheral vestibular dysfunction, or only an artifact.

## Cases presentation

### Case I

A 74 year old male with the diagnosis of probable Menière's Disease (MD) according to the recent Bárány Society diagnostic criteria for MD (4), presented 5 years of progression of recurrent vertigo attacks with concomitant ear fullness and tinnitus. The patient also has a mild broad-frequency bilateral hearing loss according to the 1997 Bureau International d'Audiophonologie (BIAP) criteria. Medical treatment with betahistine (24 mg/12h) and on demand sulpiride (50 mg) has achieved a good control of his vertigo and related symptoms. During these 5 years the patient presented periodically at a tertiary neurotology clinic to monitor his progression of probable MD.

Apart from the mild hearing loss, no significant visual impairment or other alterations were found during these 5 years on the patient otoneurotologic examination: normal ocular movements, smooth pursuit, and saccadic movement were found, along with absence of ocular misalignment on skew deviation test and no spontaneous nystagmus. Other neurological examinations and cerebral magnetic resonance images (MRI) were also normal for this patient.

#### Instrumental vestibular testing

**Figure 1:**
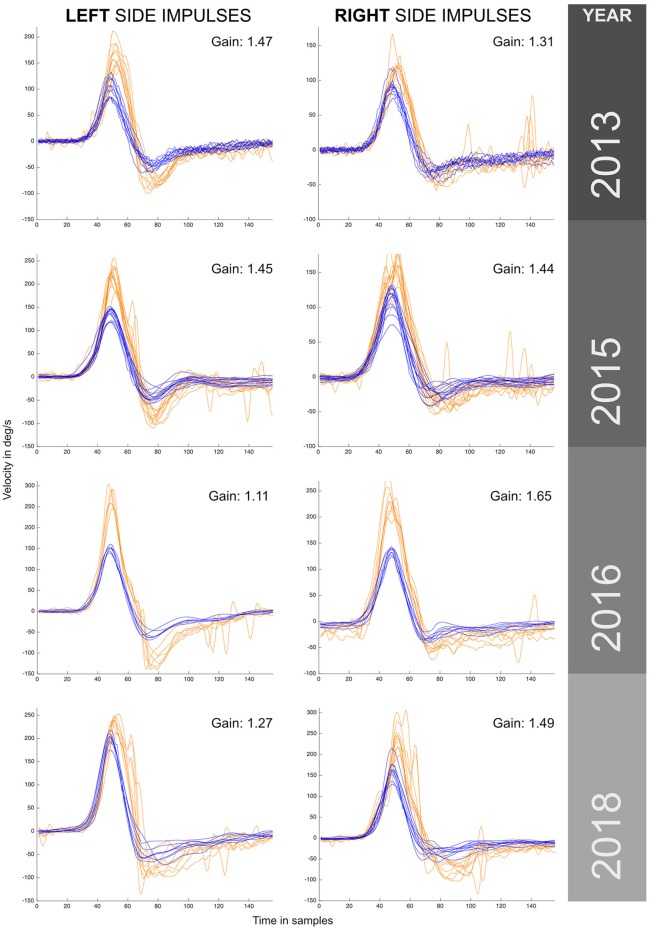
VHIT responses obtained for the same patient (case I) by the same examiner during a 5 year period with recurrent episodes of vertigo. Different versions and models from the same manufacturer were used to monitor this patient over this period. Enhanced eye responses with a higher maximum eye peak velocity and aVOR gain values above 1 were observed in all measurements. The observed enhanced eye-movement responses have asymmetrical outcomes within the same year exploration and between years, also note that in the first year (2013) enhanced eye responses were predominantly to the left, and in the last year (2018) predominantly to the right. For each vHIT plot the *x* axis represents time in samples (with a sampling frequency of ~250 Hz). The *y* axis shows the velocity in degrees/second. Orange traces show eye velocity and blue traces show head velocity.

The patient's semicircular canal function was monitored during the 5 years of development of recurrent vertigo using vHIT ICS Impulse™ devices (Otometrics A/S, Taastrup, Denmark). The vHIT explorations were always performed by a senior neurotologist, but because of the 5 year time period, different ICS Impulse™ hardware devices and software versions were used for the patient's examination: For the 2013 examination ICS Impulse™ hardware device with FireWire connection and software version 2.0 was used; for the 2015 examination the hardware was changed to ICS Impulse™ universal serial bus (USB) hardware device with software version 2.0, for the 2016 examination the software was updated to 3.0, and for the 2018 examination version 4.1 was used. The data collected during these years were exported and re-analyzed with ICS Impulse™ 4.1 version, this was made to avoid possible analysis bias in the data presented in this paper because of possible differences on gain calculation methods between the different ICS Impulse™ versions used to collect the data over time. For all vHIT tests using these different vHIT devices, the patient showed an increased aVOR gain in both horizontal canals (Figure 1). Note that the aVOR gain calculated by (version 4.1) Impulse™ software is the ratio of the area under the desaccaded eye velocity to the area under the head velocity during the impulse, and so is an area gain.

The minimum aVOR gain value was measured for the horizontal left canal in 2016 (gain of 1.11), and the maximum aVOR gain value of 1.65 was measured for the right horizontal canal in 2016. During the 5 years of testing a significantly enhanced eye velocity, with resulting enhanced aVOR gain, was always found. The value of aVOR gain fluctuated, both for tests on the same side and also one side with respect to the other. The side with the higher gain changed from the left side in 2013 to the right side in 2018.

**Figure 2:**
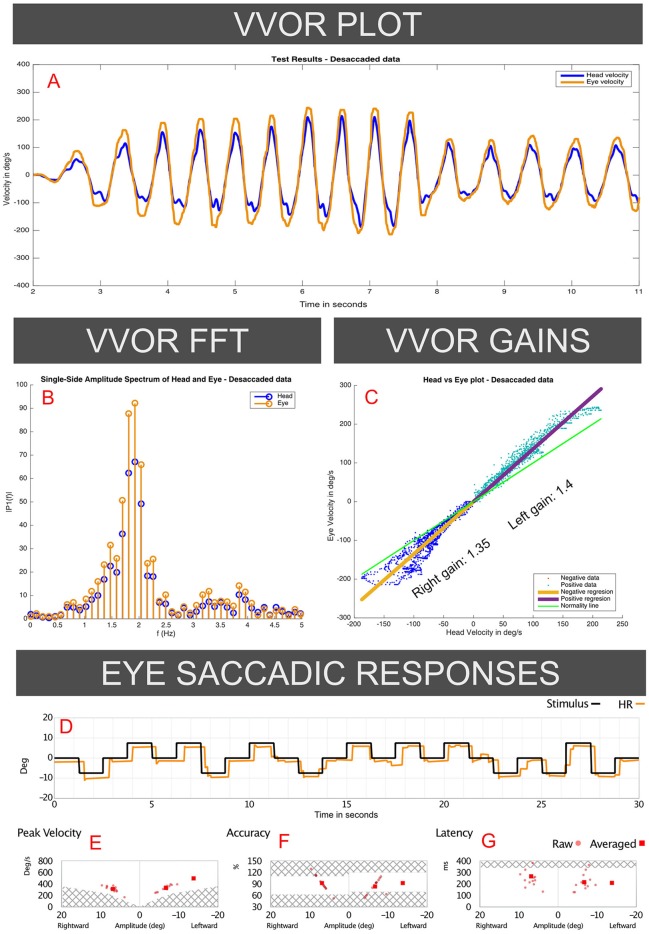
Responses to the VVOR and saccadic eye-response test recorded on the 2018 visit of the case I patient (vHIT records presented in Figure 1). Panel **A** (VVOR PLOT) shows an enhanced eye velocity in relation to head maximum velocity. In the middle panels measured gain shows gain values above 1 for rotations to both sides, observed in the linear regression between eye and head velocities (**C**: VVOR GAINS) and on fast Fourier transform (**B**: VVOR FFT). These enhanced eye responses were observed mainly, but not solely, at higher frequencies (>1.5 Hz). Panels **D–G** (EYE SACCADIC RESPONSES) show normal results on the saccadic response test measured on the same patient in the same session as the same vHIT device and calibration, without evidence of patient ocular inaccuracy or faulty calibration of the vHIT device.

In 2018 we also performed oculomotor tests including the visual-vestibular interaction (VVOR) test (5), by using the same vHIT testing equipment to obtain precise eye-movement recordings. In these oculomotor tests no position or tracking errors were found. For the saccadic eye movement test, high-velocity saccadic eye movements were recorded (Figures 2D-F).

For VVOR testing (Figures 2A-C), a bilateral enhanced eye velocity was observed. Mathematical analysis of the desaccaded VVOR eye velocity response (5) showed a positive VVOR gain value of 1.35 for the right side and 1.4 for the left side measured at ~1.8 Hz stimulation frequency. This shows that enhanced eye velocity on high velocity vHIT was accompanied by enhanced eye velocity on low-velocity VVOR testing.

### Case II

A 45 year old female with the diagnosis of 10 years' progression of definite (4) bilateral type I Menière's Disease, according to the Lopez-Escamez classification (6), with recurrent vertigo and hearing loss attacks symptomatically controlled with sulpiride (50 mg) on demand, was referred to the cochlear implants unit of a tertiary hospital center as a possible candidate for cochlear implantation, because of profound hearing loss (1997 BIAP) in the left ear and fluctuating moderate-severe (1997 BIAP) hearing loss in the right ear. The most recent vertigo episode occurred 10 days prior to her visit to the cochlear implant unit. No visual impairment or other alterations were found on otoneurotologic examination; normal ocular movements, normal smooth pursuit, and saccadic movement, absence of ocular misalignment on skew deviation test and no spontaneous nystagmus were also found, despite the recency of the last vertigo attack. Other neurological examinations and standard cerebral MRI were also normal for this patient.

**Figure 3:**
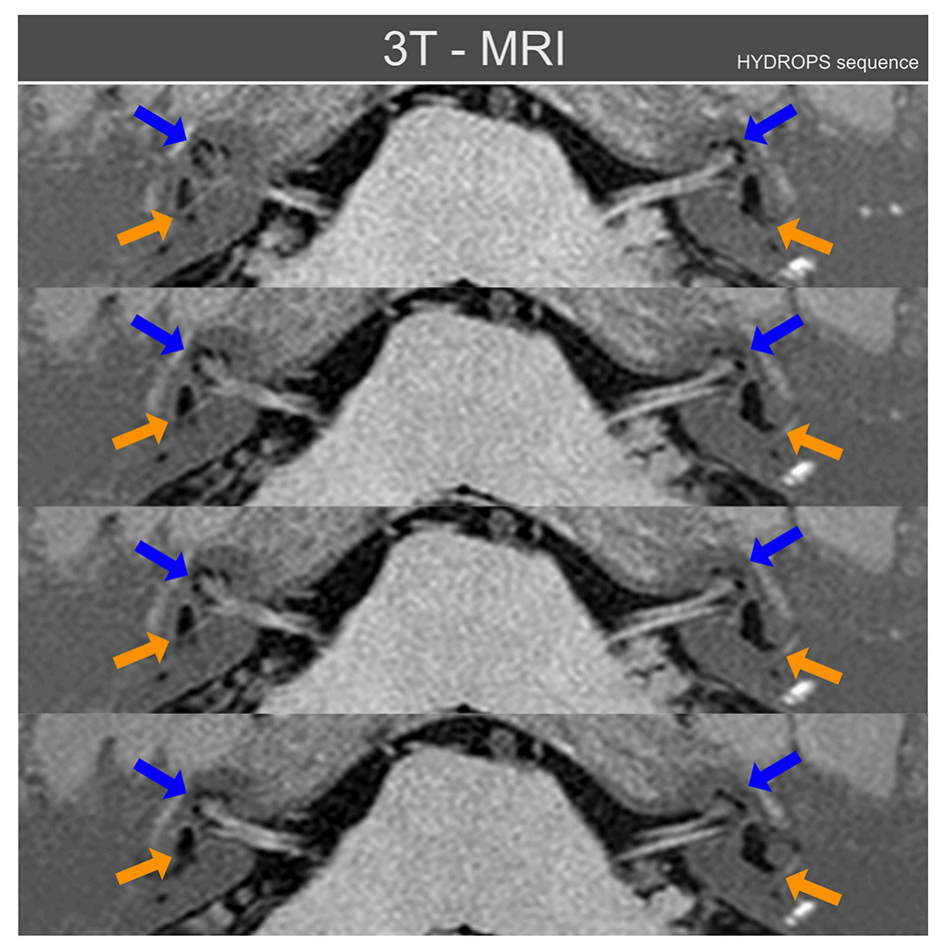
Three Tesla MRI inner ear sequences in axial projection obtained from patient case II using the MRI HYDROPS sequence reconstructed over normal MRI sequences. Blue arrows point to cochlear endolymphatic hydrops that is radiologically present on both inner ears, but mainly on left side. Orange arrows point to vestibular endolymphatic hydrops, also present on both sides but again more relevant on the left side. With the HYDROPS sequence technique the normal endolymphatic volumes are not plotted and only when there is (radiologically) significant endolymphatic hydrops does this appear on HYDROPS sequences as black color volumes. For this figure the HYDROPS sequences images were automatically added to normal MRI structures to allow an easier identification of other nearby anatomical references of inner ear MRI axial images.

In addition to the same tests as for case I, because of the bilateral MD diagnosis, the patient had received 3 months prior a 3 Tesla MRI examination of the inner ear using the HYDROPS MRI sequence (7): this sequence is based on the digital subtraction of images produced by the different time diffusion of gadolinium along the inner ear fluids. This MRI showed bilateral cochlear and vestibular endolymphatic hydrops with left side hydrops predominating, as can be observed in Figure 3.

#### Instrumental vestibular testing

**Figure 4:**
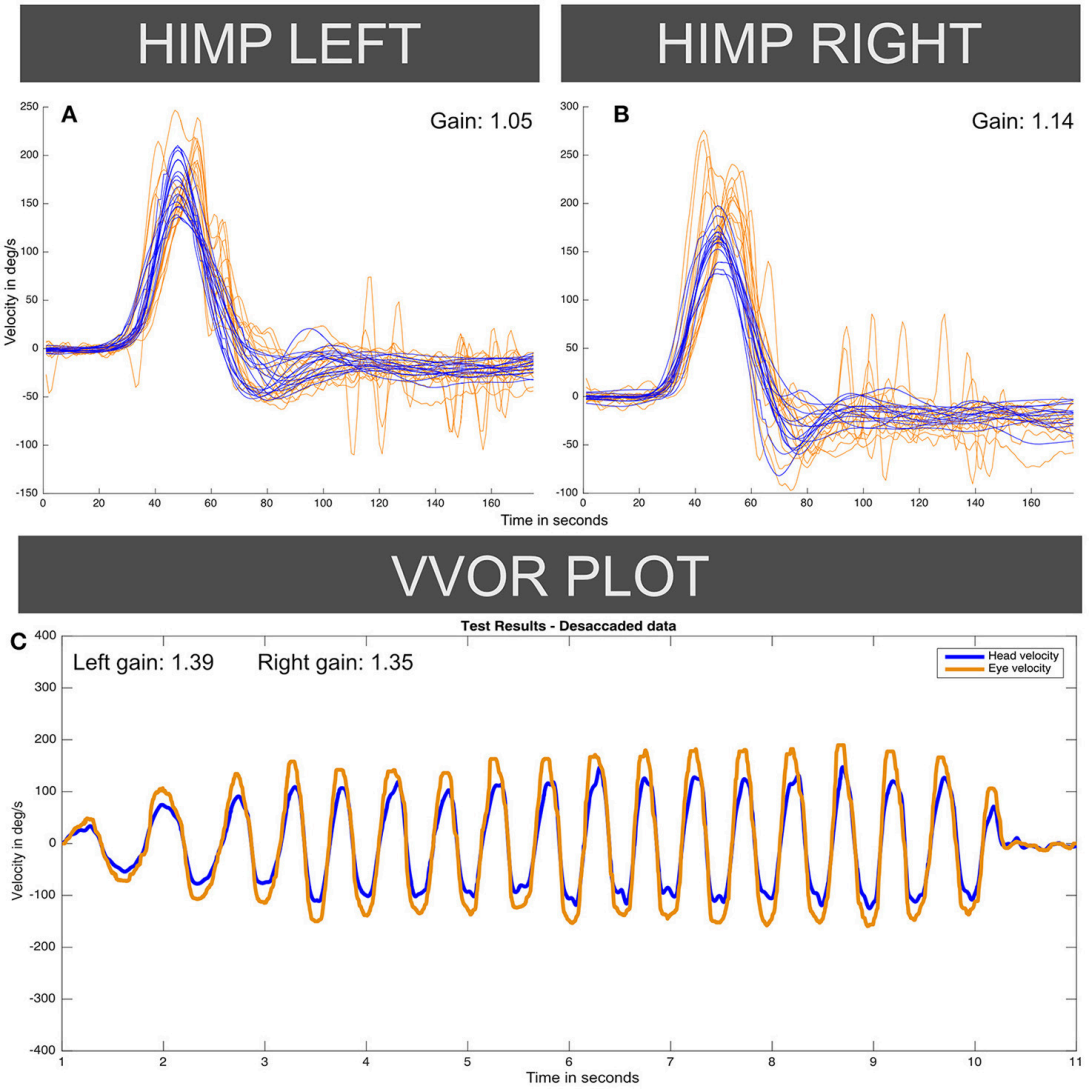
Enhanced eye-velocity responses to vHIT **(A,B)** and VVOR **(C)** obtained from the case II patient, similar to Figures 1, 2 for the case I patient. For each vHIT plot the *x* axis represents time in samples (with a sampling frequency of ~250 Hz). The *y* axis shows the velocity in degrees/second. The orange traces show eye velocity lines and blue traces show head velocity. HIMP: Head impulse (standard testing) paradigm.

The instrumental vestibular testing was performed by the same senior neurotologist using a vHIT ICS Impulse™ USB hardware version with software version 4.1: this device was a different unit from the device used in case I. Figure 4 shows enhanced vHIT eye velocity responses for both sides, with an aVOR gain value of 1.14 on right horizontal canal function test and 1.05 for left side. In this case, the vHIT calibration was repeated four times with similar enhanced gain values obtained, and a fifth calibration was done with the default system calibration parameters, also yielding similar aVOR gain values. The VVOR test was also performed on this patient using the vHIT ICS Impulse™ device, finding an enhanced eye velocity response during the VVOR test with a measured VVOR gain (5) value of 1.39 for left side and 1.35 for right side.

For both cases presented in this paper, the patients gave written consent to publish the results obtained from their clinical examinations and instrumental tests.

## Discussion

The first consideration is that this enhanced eye velocity response could be the result of an artifact or malfunction of the vHIT device. The consistency of the enhanced eye velocity responses provides a strong argument against device malfunction, because different vHIT devices were used to test these patients. The presence of some situational artifacts is unlikely again because of the consistency of the enhanced velocity eye responses in the same patient: this consistency suggests that the finding depends on an alteration of the patient's response.

Mantokoudis et al. (8) have documented artifacts in vHIT testing, and also quantified this enhanced aVOR gain response as a consistent finding obtained in 3% of the vHIT responses for patients with acute vestibular dysfunction. Those authors suggested that an inadequate calibration could be the cause of this result under this finding, but the authors of this article also recognized that enhanced gain eye responses could be observed on cerebellar dysfunction or VOR adaptation to hyperopic spectacle correction (8, 9). It is not easy to distinguish when this finding is an artifact or when it is a true sign of alteration of the head-impulse response. This ambiguity and the findings observed in the presented cases—in the first case report, the consistency over time with intra- and inter-eye response fluctuations, and in the second case the persistence of an enhanced aVOR gain value after 5 recalibrations—suggest that a calibration artifact has a low probability for explaining our results.

Another argument against calibration error as a cause of the enhanced gain is the asymmetry between sides observed on both vHIT and VVOR results obtained under the same calibration that was observed on both cases I and II. Theoretically, an incorrect calibration should affect both sides on the same test (with an inverse value). That was not the case in these patients: see Figure 1 for 2018 where left aVOR gain is 1.27 and the peak maximum eye velocity under 250 deg/s, but the right aVOR gain is 1.49 with many responses over 250 deg/s and a maximum eye velocity of 300 deg/s. It is hard to explain how a systematic calibration error can affect mainly one side in some measurements but both sides in other measurements.

Also, a systematic calibration error should affect vHIT and VVOR responses to comparably proportional extents. A systematic error should not produce one aVOR gain for one side on vHIT and a different gain on VVOR. This can be observed in Figure 4, where the gain was 1.05 for leftwards vHIT impulses but 1.14 for rightwards impulses, whereas the VVOR gains were higher on the left side than the right (1.39 vs. 1.35). Apart from a calibration error, other possible artifacts such as the patient's head spatial orientation have been previously evaluated and discarded by other authors as possibly significant modifiers of gain values on normal subjects (10).

In addition to these hypotheses, to minimize the risk of artifact in the vHIT recording of these patients, a rigorous exploration methodology was used in both cases: repeated measures were performed for each examination, always checking that goggles were tightly attached to the patient's head to avoid goggle slippage, and device calibration was always performed previous to each repetition.

With a reasonable possibility that the enhanced eye responses are not caused by an artifact, the common clinical characteristic present in both cases is a recurrent vestibular dysfunction. The aVOR gain fluctuations over time, quite similar to audiometric fluctuations observed on MD patients, registered in Case I by the vHIT and the presence in Case II of radiological evidence of bilateral endolymphatic hydrops suggests that endolymphatic hydrops could be the cause of the enhanced eye velocity responses. Curthoys et al. have also recently presented clinical evidence to support this idea that hydrops could be a possible cause for these enhanced eye velocity responses (11).

We hypothesized (and verified by fluid dynamical modeling) that from a hydro-mechanical point of view it is plausible that an increased endolymphatic volume could cause an increased effective pressure on the cupula of hydropic horizontal semicircular canal during a horizontal head impulse. This would produce an increased afferent vestibular signal depending directly on the endolymphatic hydrops magnitude. This hypothesis about the effects of an increased volume over the vestibular receptors was successfully simulated with computer models of the horizontal semicircular canal (12).

The two cases presented have some limitations. Despite recurrent vertigo being present in both cases, only the second case has the diagnosis of definite MD according to the criteria of Lopez-Escamez et al. (4). The lack of objective auditory fluctuations in the first case has limited the diagnosis of MD to a probable level. A second limitation is the relatively long time period of 3 months for the second case between the HYDROPS MRI and the vHIT test, meaning that the hydrops magnitude will not be the same as the hydrops measured on MRI. A third limitation is that despite the use of aVOR gain as a direct measurement of enhanced eye velocity responses, inspection of the some of the plots shows the absence of an evident correlation between eye and head maximum peak velocity and the aVOR gain parameter. For example, the left side plot for year 2016 (Figure 1) where the peak eye velocity response appears to be higher (300 deg/s) whereas peak head velocity was only 150 deg/s. Nevertheless, the ratio measured by the (area) aVOR gain value is only 1.11. This finding suggests that aVOR gain (using area) is not the best way to quantify enhanced eye velocity responses.

Taking all these issues into account, we suggest that enhanced eye velocity responses on head impulse testing are a clinical sign that correlates with vestibular endolymphatic hydrops, and this enhancement should be evaluated in further controlled research.

## Ethics statement

This case report was carried out in accordance with the recommendations of the local ethical committee of Hospital Universitario Donostia with written informed consent from all subjects, in accordance with the Declaration of Helsinki.

## Author contributions

IC, AB, and JR-M edited the manuscript. JR-M collected the clinical data.

### Conflict of interest statement

IC is an unpaid consultant to GN Otometrics (Taastrup, Denmark), and has received support from GN Otometrics for attendance at conferences and workshops. The remaining authors declare that the research was conducted in the absence of any commercial or financial relationships that could be construed as a potential conflict of interest.
